# Financial Toxicity Encountered in Therapeutic Radiopharmaceutical Clinical Development for Ovarian Cancer

**DOI:** 10.3390/ph13080181

**Published:** 2020-08-05

**Authors:** Charles A. Kunos, Rita Abdallah

**Affiliations:** 1 Investigational Drug Branch, Cancer Therapy Evaluation Program, Division of Cancer Treatment and Diagnosis, National Cancer Institute, Bethesda, MD 20850, USA; 2 Turning Point Services Ltd., Avon, OH 44011, USA; rita@ritaabdallah.com

**Keywords:** financial toxicity, financial hardship, financial distress, radiopharmaceutical, ovarian cancer, ovarian carcinoma, cancer survivorship

## Abstract

Financial toxicity or the debt a cancer survivor incurs from the costs of their medical cancer care is an understudied aspect in the clinical development of experimental therapeutic agents. The United States National Cancer Institute (NCI) Cancer Therapy Evaluation Program studies experimental therapeutic agents like radiopharmaceuticals in both early and late phase trials, which provide opportunities to comprehend more clearly the possible sources of financial toxicity incurred by cancer survivors. We reviewed the academic scholarship describing fiscal and social costs involved in the development of therapeutic radiopharmaceuticals. Because many ovarian cancer survivors outlive their disease through initial and, perhaps, multiple treatment courses, these women and their treatments provide context for our discussion on financial toxicity. 16 (27%) of 60 articles discuss financial toxicity incurred by women with ovarian cancer; none described financial toxicity associated with regulatory agency-approved or experimental therapeutic radiopharmaceuticals. Fiscal costs of radiopharmaceutical dose and schedule and social costs of individual productivity loss or asset expenditure arose as primary financial toxicities. The development of radiopharmaceuticals for women with ovarian cancer remains a high priority for the NCI Cancer Therapy Evaluation Program. Weighing radiopharmaceutical clinical benefit against measures of financial toxicity is challenging and warrants further study in prospective radiopharmaceutical clinical trials.

## 1. Introduction

In oncology, the term “financial toxicity” describes the fiscal debts or social distresses that a cancer survivor or, possibly, a caregiver incurs from the costs of cancer treatment [1]. Academic scholarship has identified out-of-pocket costs such as healthcare service copayments, deductibles, or coinsurance for inpatient hospital stays, outpatient medical appointments or services, and prescription drugs to manage complications as examples of financial toxicity [1]. These types of out-of-pocket costs apply both to the first course and to any subsequent courses of cancer treatment. Cancer survivors report higher financial toxicity than those persons who have not had cancer [2,3].

Experimental therapeutic cancer treatment discovery and development considers aspects of financial toxicity very late in the conventional drug-development sequence [4]. A conventional drug-development sequence engages phase 0 or 1 safety trials to inform next-step phase 2 efficacy trials and, then if warranted, involves randomized phase 3 survival trials contrasting the experimental therapeutic cancer treatment against standard treatment [4,5]. Troublesome aspects in this sequence are (i) high costs in patient, medical provider, pharmaceutical collaborator (manufacturer), or sponsor resources; (ii) complexity of trial research objectives; and (iii) an accepted confidence in the notion that observed reversible toxicity from an experimental therapeutic cancer treatment equals its clinical benefit [4,5,6]. A shift away from nonspecific chemotherapeutic or hormonal agents in oncology to more molecularly targeted and, thus, more expensive agents encourages a reevaluation of the financial toxicity of the conventional drug-development sequence.

In this reevaluation, there emerge opportunities to detect and to understand more clearly the high costs in patient, medical provider, pharmaceutical collaborator (manufacturer), or sponsor resources. A single example serves to illustrate these opportunities. The United States National Cancer Institute (NCI) and its pharmaceutical collaborators have begun a radiopharmaceutical drug-development sequence for women with advanced-stage ovarian cancers who have exhausted available therapeutic treatment options (NCT03507452). The primary objective of this first-in-human phase 1 trial is the safety of the mesothelin targeting thorium-227 (^227^Th) conjugate, in which the highest dose of the conjugate associated with tolerable organ sequelae (or its maximum tolerated dose) is the endpoint. Therapeutic radiopharmaceuticals like this need to be highly specific and have favorable metabolic and clearance factors to permit optimal tumor to normal organ irradiation [5]. The therapeutic radiopharmaceutical in this trial involves the ^227^Th radionuclide, its chelator octadentate 3,2-hydroxypyridinone (3,2-HOPO), and its ligand conjugate, the fully human anti-mesothelin monoclonal antibody anetumab ravtansine [7]. Decisions about the safety of the radiopharmaceutical are made on the basis of organ complications encountered from each component not just the whole radiopharmaceutical only. The mesothelin targeting thorium-227 conjugate is attractive for the conventional drug-development sequence because its circulating half-life of the antibody in blood and the ^227^Th radioactivity half-life are both about 19 days [7], fitting the commonplace repeatable 28-day treatment course. It is also desirable that ^227^Th radioactive decay emits five energy-rich alpha particles and two beta particles [7], which are cytotoxic to cancer cells residing anywhere in the body. Owing to limited resources in the conventional drug-development sequence, safety and efficacy data are used to make early phase development decisions but might not reliably predict late phase financial toxicity for cancer survivors. As NCI leads the drug-development sequence for radiopharmaceutical-agent combinations in the United States, it becomes increasingly essential to gather and to audit data on financial toxicity pertaining to the treatment and the medical care of patients in whom these anticancer agents are administered.

Here, we review the academic scholarship on anticipated direct and indirect fiscal costs as well as social costs related to experimental therapeutic radiopharmaceutical clinical development. Because three (62%) in every five women with ovarian cancer outlive their disease through initial and, perhaps, multiple treatment courses and thus might receive radiopharmaceuticals for relapsed disease, these women and their treatments provide context for our discussion on financial toxicity.

## 2. Results

In 2020, ovarian cancers are the tenth most common, 21,750 (1%), any-type cancer in American women [8]. However, ovarian cancers are the fifth most deadly, 13,940 (2%), in American women [8]. This discrepancy occurs because about four (79%) out of every five women with ovarian cancer at initial diagnosis have disease spreading in the abdominal cavity or elsewhere in the body. Abdominal cytoreductive surgery followed by platinum-and-taxane-based chemotherapy are a typical first course of treatment for most women with newly diagnosed disease [9,10]. When disease spreads only locally in the abdomen, up to 92 percent of such treated women survive five years [8]. When distant disease is evident, only 29 percent survive five years [8]. In 2020, there are an estimated 243,389 women living with ovarian cancer in the United States [8]. After an initial course of treatment, those women not achieving a disease-free status eventually die of disease despite subsequent courses of treatment, even when radiopharmaceuticals are tried [9]. Upon review of the academic scholarship on the topic of financial toxicity, 16 (27%) of 60 articles discuss financial toxicity incurred by women with ovarian cancer; none described financial toxicity associated with radiopharmaceuticals.

Table 1 lists the cost estimates for first and subsequent courses of ovarian cancer treatment. The list includes the regulatory agency-approved ^32^P chromic phosphate and the investigational experimental therapeutic mesothelin targeting thorium-227 conjugate. The table contrasts the cost estimates for these two radiopharmaceuticals against other anticancer agents, such as the poly (ADP-ribose) polymerase (PARP) inhibitors used in first-line maintenance or second-line treatments [11,12,13,14]. It is important to remind the reader that radiopharmaceuticals once had shown beneficial effect in this disease [15,16,17,18] and that, now that they can target disseminated disease more selectively, they are at the forefront of cancer treatment discovery [9]. Four randomized trials for the treatment of women with ovarian cancer evaluated the clinical benefit of intrabdominal infusion of the β-particle emitter ^32^P chromic phosphate [15,16,17,18]. It was well-tolerated and deemed beneficial over cytotoxic chemotherapies of the time [15,16,17,18]. However, the conventional drug-development sequence did not proceed due to (i) unwieldy ^32^P logistics, (ii) presumed inadequate agent distribution in the abdomen, and (iii) a clinical desire to study novel chemotherapy by investigators of the time. As discovered in Table 1, the direct drug cost of one cycle ^32^P chromic phosphate ($4967) for the treatment of ovarian cancer is much less than six cycles of cisplatin-paclitaxel ($22,554). A randomized trial of ^32^P chromic phosphate versus cisplatin-paclitaxel testing safety, efficacy, and cost-effectiveness is unlikely, and so, the comparison has much less meaning in our present discussion. Whether the long-term drug-development sequence for the mesothelin targeting thorium-227 conjugate would test its safety, efficacy, and cost-effectiveness versus cisplatin-paclitaxel treatment is yet to be determined.

Table 1 includes a direct cost estimate for radiopharmaceutical dosimetry ($14,175). Dosimetry is the study and measurement of absorbed radiation dose by the body. Many factors contribute to the variability of dosimetry among individuals and, thereby, encourage its formal study in early phase development of radiopharmaceuticals as it sharpens the thinking of investigators when labeling responders and nonresponders. Dosimetry is often billed once for radiopharmaceuticals, and the one-time reimbursement ($14,175) adds to the total estimated cost of the radiopharmaceutical. Here, radiopharmaceutical dosimetry exceeds expenses related to the infusion of chemotherapeutics (range, $478–1370). Cost estimates for managing hematologic or non-hematologic toxicities related to radiopharmaceutical administration have not been determined adequately to include in this article. However, as targeted radiopharmaceuticals tend to be quite selective in biodistribution, the costs for managing hematologic or non-hematologic toxicities is anticipated to be low. Cost estimates for managing hematologic or non-hematologic toxicities related to chemotherapy ($7418) or PARP inhibitor ($7273) treatment of women with ovarian cancer have been reported [19,20,21]. A conservative direct cost for a single cycle of chemotherapy ($12,107) is less than a single cycle of a PARP inhibitor ($23,451) or single cycle of a modern radiopharmaceutical ($27,547). It is too premature to calculate cost-effectiveness for radiopharmaceuticals developed for women with ovarian cancer, as trials are only in the safety portion of the conventional drug-development sequence.

### 2.1. Financial Toxicity of Radiopharmaceutical Dosage

A major limitation of radiopharmaceutical dosage is its inability to reach therapeutic levels at cancer sites because of nonspecific uptake by healthy organs or by plasma. Antibody-radiopharmaceutical conjugates, like the mesothelin targeting thorium-227 conjugate, combine highly targeted antibodies against cancer cell surface antigens with highly potent radionuclides joined by a chemical linker. The antibody itself is a very large, complex, and expensive to manufacture biomolecule when compared to small-molecule targeted drugs. High nonspecific uptake of an antibody-radiopharmaceutical conjugate might necessitate either a greater administered dosage each cycle or a co-administered ligand-blocking agonist, both raising the direct cost of each treatment cycle. The margin of safety in determining radiopharmaceutical dosage when nonspecific uptake is encountered can be achieved by either raising the individual antibody amount given or by giving less frequent dosages. The first-in-human mesothelin targeting thorium-227 conjugate phase 1 trial employs both strategies, allowing comment on the financial toxicity of both approaches.

Mesothelin anchors into normal mesothelial cell surfaces lining the abdominal peritoneum, lung pleura, eye cornea/limbus, and heart pericardium [23]. Many (76%) women with ovarian cancer have the targetable 69 kDa cell-bound mesothelin [23]. However, it turns out that many of these same women (75%) also shed a 42–44 kDa soluble mesothelin into the blood [23], which the mesothelin-targeting antibody can recognize and can bind. In one translational clinical study in women with ovarian cancer, a median blood serum level of mesothelin was 17 ng mL^−1^ (range: 0–52 ng mL^−1^) [23]. This was much higher than the healthy volunteer median blood serum level of 0 ng mL^−1^ mesothelin (range: 0–9 ng mL^−1^) [23]. Because the mesothelin targeting thorium-227 conjugate might bind to circulating mesothelin, the first-in-human phase 1 trial investigates a ^227^Th dosage of 1.5 MBq every six weeks with either a total antibody dose of 10 mg/1.5 MBq or 30 mg/1.5 MBq dose level (NCT03507452). There is an obvious a priori increase in direct drug cost of the radiopharmaceutical dosage due to an increased cost associated with antibody manufacture if the 30 mg/1.5 MBq dose level is recommended for further testing in the conventional drug-development sequence.

Circulating radiopharmaceutical radioactivity will generally be safe, might have modulatory effects on blood components like white blood cells, will present unique challenges for analytical biomarker studies, and might increase the hazard for normal organ toxicity [9]. Therefore, the costs of pharmacovigilance and medical monitoring, especially for managing hematologic complications, might be higher for radiopharmaceuticals that bind to shed cross-reacting antigens. Treatment-induced antidrug antibodies, as high as 46 percent for the anetumab ravtansine antibody [24], might introduce another drug delivery barrier that increases dosage and, thus, cost for each treatment cycle. Extrapolations made here require further study in prospective trials.

### 2.2. Financial Toxicity of Radiopharmaceutical Schedule

The schedule of radiopharmaceutical treatment depends upon cancer disease type, its spread in the body, the types of radioactive decay and anticipated sequelae of radiopharmaceuticals given, and the time necessary to recover from suffered complications of treatment. Radiopharmaceutical treatment schedules, given in cycles, are determined in trials testing clinical safety and efficacy [9]. These schedules take advantage of a cancer cell’s vulnerability to radiotherapy-induced DNA damage while simultaneously permitting normal cells in the body time to recover from that same damage [9].

There are two aspects of a radiopharmaceutical treatment schedule that impact financial toxicity: cycle duration and cycle number. Cycle duration relates to radioactive decay of the radionuclide, such as the 19-day decay of ^227^Th in its antibody-radiopharmaceutical conjugates. Cycle number depends upon the therapeutic response of visible cancer tumors and any accumulation of radiation-related toxicity. Cycle number for radiopharmaceuticals ranges from four to six cycles in order to optimize irradiation of macroscopic and microscopic disease while balancing normal organ tolerance. On the active ovarian cancer therapeutic radiopharmaceutical trial (NCT03507452), the mesothelin-targeting thorium-227 conjugate is given every six weeks for a maximum of three cycles. As an alpha-particle emitting radionuclide, ^227^Th is a highly potent cytotoxic payload that might possibly associate with fatigue, low red or white blood cell or platelet count, gastrointestinal upset, or even eye irritation [23]. A cycle duration of every six weeks, longer than the typical every 28-day cycle, might lessen direct costs of pharmacovigilance and medical monitoring. At this time, it is premature to estimate the costs for managing hematologic or nonhematologic complications. Using Table 1, the three-cycle estimate for mesothelin targeting thorium-227 conjugate direct drug ($40,116) and dosimetry ($14,175) costs totals $54,291 (in 2020 U.S. dollars), which could ultimately be less than the median six cycles ($106,200) of PARP inhibitor treatment (i.e., as used in one trial for women with recurrent ovarian cancer [13]). Alternate cycle duration or cycle number for other radionuclides, given alone or conjugated, would have higher or lower direct costs. The NCI now has trial infrastructure capacity to study alternative chemotherapy and radiopharmaceutical approaches for women with ovarian cancer [9]. For instance, an early phase 0 or 1 trial that might assess safety and efficacy of a limited number of PARP inhibitor cycles (like 2 or 4) for maximum tumor response, followed by a treatment decision window to assess changes in pretrial and posttherapy shed mesothelin as an early pre-imaging indicator of treatment response, and then followed by “switch maintenance” for a limited number (like 2 or 3) of radiopharmaceutical treatments might be studied in the near-term. Interpreting trials designed in this way are challenging, and investment in the conduct of such trials is warranted.

### 2.3. Radiopharmaceutical Economic and Social Costs

Financial toxicity associated with cancer treatment and care has been measured in material and in psychological domains [2,3]. Table 1 provides the direct economic costs incurred by ovarian cancer patients for treatment of recurrent disease. Treatment and care are most often paid by third-party insurers, but there are other healthcare service copayments, deductibles, and coinsurance costs that patients and caregivers are billed for inpatient hospital stays, outpatient medical appointments or services, and prescription drugs used to manage complications. These latter indirect fiscal debts, as reported by cancer survivors, take the material form of personal debt against bank credit or home equity lines of credit, borrowed money from family or from banks, asset liquidation such as the selling or refinancing a home or moving to a smaller residence, or bankruptcy and the financial protections that it provides. In one survey study of 963 respondents, two percent filed for bankruptcy and 16 percent reported some for financial toxicity in the material domain [2].

Social financial toxicity manifests in the psychological domain as excessive worry about large out-of-pocket medical bills. In one survey of 4753 cancer survivors, 25 percent cited issues about paying their out-of-pocket medical bills while 33 percent of respondents reported worry about medical bills [3]. Social financial toxicity was more common among the uninsured (50%) than among those with public (36%) or private (33%) insurance coverage [3]. The number of cancer survivors continues to grow, and many of those survivors admit struggle to pay for their cancer and medical care. The NCI is in a strong position to study sustainable approaches for patients and medical providers to reduce out-of-pocket treatment and medical care costs as a component of cancer care.

### 2.4. Radiopharmaceutical Indirect Costs

The term “indirect costs” in oncology describes the costs incurred from the cessation or reduction of work productivity from consequential morbidity or mortality associated with a given cancer disease. Examples of indirect costs consist of work loss, worker replacement, and reduced productivity from cancer itself or its medical monitoring such as attending outpatient medical appointments or services. This applies equally to the cancer patient and their medical caregivers. In one survey, cancer survivors identified higher annual out-of-pocket expenditures than did patients without a cancer history [3]. Out-of-pocket spending was highest among survivors who were not working (4%) followed by those who were working part-time (3%) and those who were working full-time (1%) [3]. Medical monitoring and pharmacovigilance associated with patient-provider inpatient hospital stays, outpatient medical appointments or services, and prescription drugs managing treatment complications adds time away from work and lowers individual productivity. Survivors employed at or after diagnosis and who then took extended paid or unpaid leave or switched to part time due to cancer were most likely to report multiple material domains of financial toxicity (34%) as compared with those who were employed but did not take extended leave or switch to part time (27% [2]). Indirect costs associated with caregivers was not captured well in our review, and therefore, no comment can be made. Further research is needed on incurred costs to caregivers of cancer survivors during and after treatment.

## 3. Discussion

Our analysis is the first to our knowledge to discuss the financial toxicity, fiscal or social, associated with therapeutic radiopharmaceuticals over the last 30 years of the conventional drug-development sequence. Our findings show that radiopharmaceutical dosage and schedule, medical monitoring and pharmacovigilance, as well as patient and caregiver productivity loss or asset expenditure contribute to financial toxicity encountered in the conventional drug-development sequence. Our opinion is that patient, medical providers, pharmaceutical collaborators (manufacturers), and sponsors alike should invest in targeted radiopharmaceutical development. The NCI and its Cancer Therapy Evaluation Program (CTEP), now and in the foreseeable future, have cost-effective and efficient pathways for a radiopharmaceutical drug-development sequence and should invest in research to expand the knowledge of financial toxicity for radiopharmaceuticals.

Higher financial toxicity in oncology may be explained by multiple factors. First, the recovery of clinical development costs drives manufacturers to set relatively high prices for drugs or radiopharmaceuticals used for a short period of time like two or three cycles (Table 1). This likely explains why the two radiopharmaceuticals used in the care of women with ovarian cancer in whom all other treatments are exhausted are priced as they are. In this clinical scenario, the duration of radiopharmaceutical treatment, where these agents are commonly used for weeks or months, is usually shorter than in other courses of treatment with chemotherapy or small-molecule drugs, which may be used for months or years. Second, available regulatory agency-approved therapeutic options are much fewer for cancer indications than in other medical disease indications, which makes patients, medical providers, pharmaceutical collaborators (manufacturers), and sponsors less responsive to the direct prices of radiopharmaceuticals and their indirect costs. Third, it is our contention that payers of cancer treatment have restricted means to reduce market prices for cancer drugs or radiopharmaceuticals because of the high cost of preclinical and clinical development and the capitalistic nature of the United States economy.

This article intended to uncover fiscal or social factors contributing to financial toxicity encountered by cancer survivors in the radiopharmaceutical drug-development sequence and did not aim to fault any means pharmaceutical collaborators, manufacturers, distributors, or medical providers in determining the asking price for their agents or services. Also, our study could not assess radiopharmaceutical value studied in terms of cost-effectiveness or clinical outcomes because modern agents remain in the early phases of the drug-development sequences. When pricing drugs or radiopharmaceuticals in this article, our research relied on published data or discoverable average wholesale prices, which do not account for rebates or other mechanisms of manufacturer financial assistance. Overall, we find that unique treatment-related fiscal and social distresses arise, and these distresses can be best valued and discussed in the setting of radiopharmaceutical clinical use for women with ovarian cancer. Further research is forthcoming.

## 4. Materials and Methods

For this article, a systematic search was done of the academic scholarship in PubMed (www.pubmed.gov) available between January 1966 and June 2020 or in Google Scholar (scholar.google.com) available between January 1995 and June 2020. Search terms included “financial toxicity,” “financial distress,” “financial hardship,” or “financial burden” and “ovarian cancer” or “ovarian carcinoma.” Articles available in the English language were reviewed for overarching research hypotheses, result domains, and data interpretations.

Cost information found in Table 1 was abstracted either from published studies [19,20,21], NCI databases or experimental therapeutic clinical trial network infusion centers, or the Centers for Medicare and Medicaid (CMS) average sales price file (www.cms.gov/medicare/medicare-part-b-drug-average-sales-price/2020-asp-drug-pricing-files; accessed 4 June 2020). The CMS file only lists drugs or radiopharmaceuticals with an approved indication from the United States Food and Drug Administration (FDA). Data from the CMS file was cross-referenced with published data [19,20,21] and adjusted by costs billed by NCI experimental therapeutic clinical trial network cost centers, when applicable. Costs for investigational experimental therapeutic agents were estimated (+6% allowable adjustment) from Current Procedural Terminology (CPT) code J3490 ($24.06 mg^−1^) for anetumab ravtansine, from CPT code A9564 ($331 mCi^−1^) for ^32^P phosphate, and from CPT code A9606 ($128 µCi^−1^) for the mesothelin targeting thorium-227 conjugate. Individualized dosimetry costs are abstracted from NCI resources and reflect estimates of radiotherapy reimbursement and not billable charges [22].

## 5. Conclusions

In conclusion, this article provides one perspective on financial toxicity possibly encountered in a radiopharmaceutical drug-development sequence. The article used current experience from clinical use of a mesothelin targeting antibody-radiopharmaceutical conjugate for the treatment of women with ovarian cancer who have exhausted all other forms of treatment. The article intended to sharpen the focus and thoughts about financial toxicity for new radiopharmaceuticals. Important overarching topics related to regulatory safety and pharmacovigilance are not discussed here. Guidance for some of these topics are found elsewhere [25]. Consideration of measures of financial toxicity encountered in the radiopharmaceutical drug-development sequence for women with ovarian cancer should be done in next-step clinical trials.

## Figures and Tables

**Table 1:** Estimated costs for select drugs or radiopharmaceuticals used to treat ovarian carcinoma, per cycle ^1^.

Agent	Drug	Dose	Drug	Infusion	Total
Platinum-based	Carboplatin	300 mg m^−2^ BSA IV QM	$1702	$449	$2151
therapies	Cisplatin	75 mg m^−2^ BSA IV QM	$1145	$481	$1626
	Paclitaxel	175 mg m^−2^ BSA for 3 h QM	$2614	$449	$3063
Non-platinum	PL Doxorubicin	50 mg m^−2^ BSA IV for 1 h QM	$4287	$478	$4765
based therapies	Abraxane	100 mg m^−2^ BSA IV d1,8,15 QM	$5079	$478	$5557
	Pemetrexed	900 mg m^−2^ BSA IV for 10 m Q3W	$9560	$770	$10,330
	Topotecan	1.5 mg m^−2^ BSA IV QD for 5 d Q3W	$283	$1452	$1735
Anti-angiogenesis-based therapies	Bevacizumab + Paclitaxel	10 mg kg^−1^ IV Q2W plus 80 mg m^−2^ BSA IV d1,8,15,22 QM	$9608	$1171	$10,779
	Bevacizumab + PL Doxorubicin	10 mg kg^−1^ IV Q2W plus 40 mg m^−2^ BSA IV for 1 h QM	$12,936	$791	$13,774
	Bevacizumab + Topotecan	10 mg kg^−1^ IV Q2W plus 4 mg m^−2^ BSA IV d1,8,15 QM	$9958	$1171	$11,129
PARPi	Niraparib	300 mg PO QD	$17,700	$0	$17,700
	Olaparib	400 mg PO BID	$16,178	$0	$16,178
	Rucaparib	600 mg PO BID	$16,488	$0	$16,488
Antibody-drug conjugate	Anetumab ravtansine	2.2 mg kg^−1^ IV for 1 h QW	$3176	$864	$4040
Radiopharmaceutical	^32^P phosphate	15 mCi IP QM single dose	$4967	$14,175 ^2^	$19,142
	^227^Th conjugate	7 MBq IV Q6W single dose	$13,372	$14,175 ^2^	$27,547

^1^ In United States Dollars ($). ^2^ Estimate includes a one-time, first-cycle individualized dosimetry charge [22]. Abbreviations: BID: twice daily; BSA: body surface area; d: days; h: hours; IP: intraperitoneal; IV: intravenous; m: minutes; PARPi: poly (ADP-ribose) polymerase inhibitors; PL: pegylated liposomal; PO: oral; Q2W: every 2 weeks; Q3W: every 3 weeks; Q6W: every 6 weeks; QD: daily; QM: monthly; QW: weekly.
